# The degeneration of locus coeruleus occurring during Alzheimer’s disease clinical progression: a neuroimaging follow-up investigation

**DOI:** 10.1007/s00429-024-02797-1

**Published:** 2024-04-16

**Authors:** Alessandro Galgani, Francesco Lombardo, Francesca Frijia, Nicola Martini, Gloria Tognoni, Nicola Pavese, Filippo Sean Giorgi

**Affiliations:** 1 Department of Translational Research and of New Surgical and Medical Technologies, University of Pisa, Via Roma 55, Pisa, 56126 Italy; 2 Department of Radiology, Fondazione Toscana G. Monasterio, Pisa, Italy; 3 Bioengineering Unit, Fondazione Toscana G. Monasterio, Pisa, Italy; 4 Department of Clinical and Experimental Medicine, University of Pisa, Pisa, Italy; 5 Clinical Ageing Research Unit, Newcastle University, Newcastle upon Tyne, UK; 6 Institute of Clinical Medicine, PET Centre, Aarhus University, Aarhus, Denmark

**Keywords:** Locus Coeruleus, Alzheimer’s Disease, Magnetic resonance imaging, Mild cognitive impairment, Noradrenaline, Neurodegeneration

## Abstract

The noradrenergic nucleus Locus Coeruleus (LC) is precociously involved in Alzheimer’s Disease (AD) pathology, and its degeneration progresses during the course of the disease. Using Magnetic Resonance Imaging (MRI), researchers showed also in vivo in patients the disruption of LC, which can be observed both in Mild Cognitively Impaired individuals and AD demented patients. In this study, we report the results of a follow-up neuroradiological assessment, in which we evaluated the LC degeneration overtime in a group of cognitively impaired patients, submitted to MRI both at baseline and at the end of a 2.5-year follow-up. We found that a progressive LC disruption can be observed also in vivo, involving the entire nucleus and associated with clinical diagnosis. Our findings parallel neuropathological ones, which showed a continuous increase of neuronal death and volumetric atrophy within the LC with the progression of Braak’s stages for neurofibrillary pathology. This supports the reliability of MRI as a tool for exploring the integrity of the central noradrenergic system in neurodegenerative disorders.

**Supplementary Information:**

The online version contains supplementary material available at 10.1007/s00429-024-02797-1.

## Introduction

In the last few years, the noradrenergic (NA) nucleus Locus Coeruleus (LC) has been receiving growing attention in the field of Alzheimer’s Disease (AD). The increase of interest in this small pontine structure is due to neuropathological studies, which clearly showed that LC pathological alteration is the first AD-related feature detectable in the ageing human brain (Braak et al. 2011), and that a severe and marked degeneration of the nucleus already occurs at the onset of AD-related cognitive symptoms (Kelly et al. 2017). Moreover, plenty of experimental evidence has linked LC functional impairment to pathogenetic mechanisms of AD, ranging from aberrant microglial activity to increased amyloid accumulation (Heneka et al. 2006; Beardmore et al. 2021). In this context, the development of LC Magnetic Resonance Imaging (LC-MRI)(Galgani et al. 2020) allowed to confirm the early degeneration of this nucleus in AD patients (Betts et al. 2019; Galgani et al. 2023). MRI was validated as a tool to evaluate in vivo the integrity of LC, as shown by Keren and colleagues in 2015, who revealed a direct association between LC-MRI signal intensity and the number of NA cells in the LC of the same brainstem sample (Keren et al. 2015). The link with AD was then strengthened by subsequent investigations, which highlighted the association occurring with AD-related tau and amyloid pathology, both assessed through Positron Emission Tomography (PET) (Dahl et al. 2021; Jacobs et al. 2021, 2023; Calarco et al. 2022; Van Egroo et al. 2022). In a previous study, we performed LC-MRI in a population belonging to the AD clinical *continuum* (Galgani et al. 2023). Using a template-based approach for the analysis of LC images, we found that the early loss of integrity of the rostral part of this nucleus predicts the progression toward overt dementia in subjects affected by Mild Cognitive Impairment (MCI) at baseline. Our results are in line with anatomical data obtained in healthy subjects, showing that NA neurons projecting to limbic areas - the first brain regions affected by AD-related Tau pathology - are placed mainly in the rostral part of LC (Schwarz and Luo 2015). Those results also fit with very recent findings obtained with ultra-High field MRI scan in aged subjects (Van Egroo et al. 2023), showing an association of plasma hyperphosphorylated tau and lower dorsal-rostral LC integrity starting in midlife.

LC degeneration might represent a dynamic phenomenon, which may progress in parallel with AD progression. This is in line with a recent post-mortem analysis of the degeneration of the LC across neurofibrillary pathology-related Braak stages (BB stage-Braak et al. 2011) in a cohort of AD patients and aged controls, in which for the first time stereological analysis was used to precisely estimate LC neuronal number, showing progressive LC neuron loss and atrophy along BB stages, from I to VI (Theofilas et al. 2017). However, to the best of our knowledge such a temporal progression of LC neuronal loss in pathological ageing has not been tested thus far in vivo: MRI allows indeed to assess at different time points, in each subject the variation of LC signal.

Thus, in this study involving subjects submitted to a clinical and LC-MRI assessment at two different time points, we aimed to investigate if MRI could detect the progressive degeneration of LC during AD evolution, and whether the extent of any possible time-dependent alterations was associated with the clinical progression, in line with the abovementioned post-mortem data.

## Methods

A sub-group of the patients recruited in a previous study (Galgani et al. 2023) who underwent a 2.5 years clinical follow-up, also consented to undergo a parallel LC-MRI follow-up. Patients were clinically diagnosed as amnestic MCI(Albert et al. 2011) or Alzheimer’s Disease Dementia(McKhann et al. 2011) at baseline (T0) upon a thorough neurological and neuropsychological assessment (Galgani et al. 2023). Then, they underwent a 2.5-year clinical follow-up, to monitor the progression of cognitive symptoms and classify MCI individuals as converter (cMCI) or non-converter (ncMCI), based on whether they converted or not to dementia. At the end of follow-up (T1), patients included in the present study were submitted to a second neurological and neuropsychological evaluation, and to a second LC-MRI scan, using the same 3Tesla MR-Unit (GE Excite HDx, GE, USA). According to Helsinki declaration, patients gave their written informed consent, and the study was approved by the Ethics Committee of Area Vasta Nord-Ovest of Tuscany Region Health System (#1203, PE-2013-02349574). For details on patients inclusion and selection criteria, please see Supplem. Methods&Results S1.

### Brain MRI protocol and post-processing

Both T0 and T1 brain MRI scans were performed using a 3.0 Tesla MR unit (Excite HDx, General Electric) with an eight-channel phased- array head coil. As described in (Galgani et al. 2023), the protocol included: two-dimensional (2D) fluid-attenuated inversion recovery, T2* gradient recall echo, and spin echo T1- and fast spin echo (FSE) T2-weighted with fat saturation and diffusion- weighted imaging. Furthermore, whole brain 3D-Fast-SPGR T1-weighted images were obtained: TR 10.7ms; TE 4.9ms; FOV 256 × 256 mm; matrix size 256 × 256; isotropic voxel 1 mm; NEX 1; acquisition time: 5.50 min.

A voxel-based morphometry (VBM) and a ROI-based hippocampal volume analysis were also performed to assess cortical atrophy; for details, please see Supplem. Methods & Results S1.

### LC imaging

The LC-sensitive sequence was acquired along the oblique axial plane, perpendicular to the fourth ventricle floor, covering an area from the inferior border of the pons to the posterior commissure. We used a 2D-FSE T1-weighted sequence: TR 600ms; TE 14ms; flip angle 90°; echo train length 2; NEX 5; matrix size 512 × 384; FOV 200 × 200 mm; pixel size 0.39 × 0.52 mm; 12 contiguous slices, slice thickness 2.2 mm, slice gap 0; acquisition time 14.29 min.

**Fig. 1:**
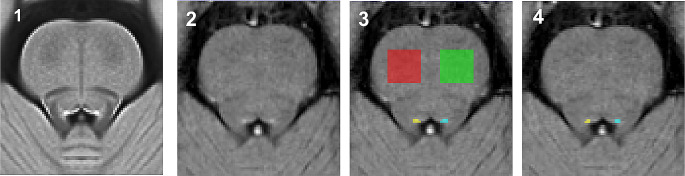
Template-based method for LC-MRI parameters estimation. LC images acquired in included subjects were warped together to build a brainstem template; signal intensity threshold is calculated, and LC mask extracted (1). Please note that the latter step refers to the whole cohort, published in Galgani et al. 2023. Then, the single patient LC sequence was warped into the brainstem template (2). LC mask was superimposed and LC_CR_ parameter was extrapolated (3). A subject-specific threshold was calculated to compute the LC_VOX_ parameter (4)

After the acquisition, LC images were visually inspected in order to exclude cases showing motion or technical artifacts. LC-MRI parameters were extracted using the same template-based approach developed in the previous studies (Fig. 1) (Giorgi et al. 2022; Galgani et al. 2023). Briefly, first, we created a common space for 3D anatomical MRI using only T0 MRI images of the healthy and cognitively intact subjects belonging to the included cohort (Galgani et al. 2023). Each individual 3D dataset was interpolated to an isotropic resolution, underwent a nonuniform intensity correction, and was then subjected to a multiresolution iterative registration. Second, the native 2D space LC sequences of the same healthy subjects were warped into the 3D common space using the transformation matrices and warping fields obtained during its creation. This resulted in a template space of the brainstem area where LC sequence acquisition was performed (isotropic resolution 0.5 mm). The template exhibited a high signal-to-noise ratio with a markedly intense spot just below the floor of the fourth ventricle. A semiautomatic thresholding procedure was applied, involving the placement of two reference regions (ROIs) in the ventral pons. The mean intensity (µ_ROI_) and standard deviation (σ_ROI_) of these ROIs were calculated, and only the voxel in the whole template exceeding µ_ROI_ by four times σ_ROI_ were selected. The identified voxels were pooled together to build the LC mask, which was then further manually refined (Galgani et al. 2023).

Both T0 and T1 MRI scans were then warped into the brainstem template and the above-described LC mask was applied. Then, LC parameters were calculated, namely the LC Contrast Ratio (LC_CR_), which is considered a proxy of neuronal density, and LC-belonging voxels (LC_VOX_), which represents an indirect estimation of the LC volume. LC parameters were extrapolated for the two hemispheres, both combined (LC complex) and as separate ones (Left and Right LC). A subregional analysis was also performed along the rostro-caudal axis by dividing the LC mask into two equally sized parts: the rostral and the caudal one.

### Statistical analysis

Outliers trespassing the Tukey’s outer fence were not included in the analysis. Normality of variables and residuals, as well as heteroscedasticity, were checked visually and through Shapiro-Wilk test. Given non-gaussian distribution, we used Wilcoxon paired-sample test to assess the variation between baseline and follow-up MRI assessments parameters. Cohen’s d coefficient was calculated for each parameter to estimate the effect size and to compare the degree of LC variation over time across diagnostic groups. Linear Mixed Models (LMMs) were built to better explore the effect of the diagnosis at the end of the follow-up (ncMCI, cMCI and ADD) on LC-MRI parameters variation over time. LC_CR_ or LC_VOX_ of the LC complex, either entire or divided into its rostral and caudal parts, as well as of the separated Left and Right LCs, were taken as dependent variables, while time and diagnosis were considered as fixed factors. Estimated Marginal Means (EMMs) were calculated for the factor time, diagnosis and time*diagnosis. Multiple comparison adjustment was addressed using False Discovery Rate (FDR) correction. The level of significance was set at *p* < 0.05. SPSS Version 25 was used to perform the statistical analysis, while plots were produced with Prism GraphPad 9.0.

## Results

### Included patients

Sixty patients were eventually recruited to take part to this study. Three of them were then excluded from the final analysis due to the occurrence of motion artifacts either in the baseline (*N* = 2) or in the follow-up (*N* = 1) scan. A final cohort of 57 subjects was included in the study, 12 of them being diagnosed as ADD and 45 as MCI at T0. Nineteen of the MCI individuals converted to dementia during the follow-up (cMCI), while the remaining 26 did not show a clinically relevant progression of the cognitive disorder (ncMCI). Table 1 reports the detailed demographical and clinical description of the study group. For the results of VBM and hippocampal volume analysis, please see Supplem. Methods&Results S1.

**Table 1:** Demographic, clinical and genetic description of study population

		MCI	ncMCI	cMCI	ADD	p-value
Subjects		45	26	19	12	*-*
Age	Mean ± SD	72.9 ± 4.3	72.0 ± 3.9	74.1 ± 4.5	70.8 ± 7.0	*0.163* ^*^
Sex	Males %	47%	58%	32%	42%	*0.211* ^*+*^
APOE genotype	ε4 carrier %	40%	42%	37%	25%	*0.589* ^*+*^
MMSE baseline	Mean ± SD	24.1 ± 2.6	24.8 ± 2.3	23.2 ± 2.6	20.7 ± 5.9	*0.022* ^*a*^
MMSE follow-up	Mean ± SD	20.9 ± 5.0	23.4 ± 3.5	17.6 ± 5.0	13.3 ± 5.0	*0.001* ^*b*^
CDR baseline	Mean ± SD	0.41 ± 0.19	0.42 ± 0.18	0.39 ± 0.21	1.00 ± 0.00	*0.022* ^*c*^
CDR follow-up	Mean ± SD	0.77 ± 0.42	0.48 ± 0.09	1.16 ± 0.37	2.33 ± 0.49	*0.001* ^*d*^
Ed. period (years)	Mean ± SD	8.7 ± 4.0	9.7 ± 3.9	7.3 ± 3.8	8.1 ± 3.8	0.612 *

ADD: patients affected by Alzheimer’s dementia at baseline; MCI: Mild Cognitive Impairment; cMCI: MCI patients which converted to dementia during follow-up; ncMCI: MCI patients which did not convert to dementia during follow-up; MMSE: Mini-Mental State Examination

^*^ Kruskal-Wallis test significance; ^**+**^Chi-square pair-wise comparisons significance; ^a^ Mann-Whitney pair-wise comparisons regarding MMSE at baseline: ncMCI vs. cMCI (*p* = 0.279), cMCI vs. ADD (*p* = 0.747), ncMCI > ADD (*p* = 0.023); ^b^ Mann-Whitney pair-wise comparisons regarding MMSE at 2.5 years follow-up: ncMCI > cMCI (*p* = 0.001), cMCI vs. ADD (*p* = 0.456), ncMCI > ADD (*p* = 0.001); ^c^ Mann-Whitney pair-wise comparisons regarding CDR at baseline: ncMCI vs. cMCI (*p* = 1.000), cMCI vs. ADD (*p* < 0.001), ncMCI > ADD (*p* < 0.001); ^d^ Mann-Whitney pair-wise comparisons regarding CDR at 2.5 years follow-up: ncMCI < cMCI (*p* < 0.001), cMCI < ADD (*p* = 0.048), ncMCI < ADD (*p* < 0.001)

### LC-MRI parameters variation over time

**Fig. 2:**
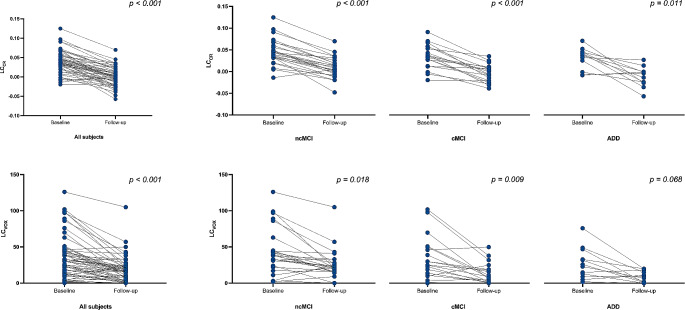
LC-MRI parameters reduction over-time. The charts show the reduction in both the LC parameters (LC_CR_ and LC_VOX_) occurring between baseline and follow-up MRI scan. Reported p-values are adjusted for FDR multiple comparison correction and refers to Wilcoxon paired-samples test performed. ADD: patients affected by Alzheimer’s Disease Dementia at baseline; cMCI: Mild Cognitive Impairment subjects which converted to dementia during follow-up; ncMCI: Mild Cognitive Impairment subjects which did not convert to dementia during follow-up

Wilcoxon signed rank test confirmed that both LC-MRI parameters were reduced during follow-up across the whole population (*p* < 0.001), and in all the diagnostic groups, except for LC_VOX_ in the ADD group (Fig. 2; Table 2). Moreover, an association with the clinical diagnosis was found, with ncMCI showing a milder LC signal decrease over time (Cohen’s d = 1.37, *p* < 0.001 for LC_CR_ and Cohen’s d = 0.67, *p* = 0.001 for LC_VOX_) than cMCI (Cohen’s d = 1.61, *p* < 0.001 for LC_CR_ and Cohen’s d = 0.91, *p* = 0.009 for LC_VOX_) and ADD (Cohen’s d = 1.81, *p* = 0.011 for LC_CR_ and Cohen’s d = 1.15, p = *ns* for LC_VOX_). LMMs confirmed the reduction of LC-MRI parameters over time (see Supplem. Table 2), but not the association with the diagnosis; even though EMMs calculated for the factor time*diagnosis showed a trend, they did not reach statistical significance (Supplem. Table 2). Pooling together cMCI and ADD (which at T1 were all diagnosed as ADD), provided similar results (data not shown).Topographically, the reduction of LC signal involved the entire nucleus, without any specific subregional asymmetry (Table 2, Supplem. Tables 5 and 6).

**Table 2:** Wilcoxon paired samples tests

	T	All subjects					ncMCI					cMCI					ADD				
		M	SD	p	ES	%	M	SD	p	ES	%	M	SD	p	ES	%	M	SD	p	ES	%
**Complex**																					
LC_CR_	T0	0.0390	0.0283	< 0.001*	1.490	-100%	0.0457	0.0297	< 0.001*	1.370	-81%	0.0350	0.0278	< 0.001*	1.610	-112%	0.0307	0.0247	0.011*	1.820	-140%
	T1	0.0000	0.0237				0.0089	0.0237				-0.0043	0.0204				-0.0124	0.0228			
LC_VOX_	T0	37.09	29.51	< 0.001*	0.790	-52%	43.69	31.82	0.001*	0.670	-41%	34.11	29.74	0.009*	0.910	-62%	27.50	21.60	**0.068**	1.150	-68%
	T1	17.93	17.81				25.85	20.21				12.89	14.38				8.75	8.01			
**Rostral part**																					
LC_CR_	T0	0.0363	0.0267	< 0.001*	1.390	-99%	0.0423	0.0289	< 0.001*	1.180	-76%	0.0327	0.0262	< 0.001*	1.700	-122%	0.0288	0.0206	0.015*	1.660	-130%
	T1	0.0003	0.0250				0.0101	0.0254				-0.0073	0.0205				-0.0088	0.0246			
LC_VOX_	T0	18.39	16.85	< 0.001*	0.710	-55%	22.31	19.07	0.003*	0.590	-44%	16.53	16.07	0.011*	0.970	-71%	12.83	10.99	**0.062**	0.930	-62%
	T1	8.30	10.96				12.42	14.27				4.84	5.43				4.83	5.15			
**Caudal Part**																					
LC_CR_	T0	0.0433	0.0358	< 0.001*	1.250	-95%	0.0510	0.0370	< 0.001*	1.190	-79%	0.0389	0.0350	< 0.001*	1.250	-98%	0.0333	0.0334	0.018*	1.540	-144%
	T1	0.0022	0.0296				0.0109	0.0304				0.0008	0.0250				-0.0147	0.0289			
LC_VOX_	T0	22.11	18.15	< 0.001*	0.700	-49%	25.58	18.42	0.004*	0.600	-36%	20.68	18.81	0.009*	0.750	-57%	16.83	16.25	**0.063**	1.000	-74%
	T1	11.35	11.70				16.31	11.72				8.95	11.66				4.42	6.42			
**Left LC**																					
LC_CR_	T0	0.0460	0.0329	< 0.001*	1.280	-82%	0.0553	0.0321	< 0.001*	1.310	-68%	0.0391	0.0341	0.001*	1.270	-95%	0.0367	0.0300	0.024*	1.380	-103%
	T1	0.0085	0.0253				0.0177	0.0248				0.0020	0.0233				-0.0011	0.0244			
LC_VOX_	T0	22.16	18.09	< 0.001*	0.740	-50%	27.35	18.42	0.003*	0.720	-41%	19.05	18.42	0.009*	0.850	-64%	15.83	14.70	**0.061**	0.820	-58%
	T1	11.11	10.72				16.27	11.68				6.89	8.64				6.58	6.07			
**Right LC**																					
LC_CR_	T0	0.0319	0.0279	< 0.001*	1.500	-127%	0.0361	0.0313	< 0.001*	1.260	-100%	0.0308	0.0263	< 0.001*	1.720	-135%	0.0247	0.0230	0.015*	1.950	-196%
	T1	-0.0085	0.0258				0.0002	0.0255				-0.0107	0.0217				-0.0237	0.0267			
LC_VOX_	T0	14.93	14.41	< 0.001*	0.670	-54%	16.35	15.95	0.009*	0.500	-41%	15.05	15.05	0.011*	0.750	-60%	11.67	9.59	**0.066**	1.280	-81%
	T1	6.82	9.13				9.58	10.53				6.00	8.25				2.17	4.28			

Absolute values of LC-MRI parameters are reported at T0 (baseline) and T1 (end of the 2.5 years follow-up) for the whole cohort and each diagnostic group. The FDR corrected p-values (p) of performed Wilcoxon paired samples test are also reported, alongside with the relative Cohen’s d or effect size (ES). In the column named (%), percentual reduction of LC-MRI parameters between T0 and T1 assessments can be found. ADD: patients affected by Alzheimer’s Disease Dementia at baseline; M: Mean; MCI: Mild Cognitive Impairment; cMCI: MCI patients which converted to dementia during follow-up; ncMCI: MCI patients which did not convert to dementia during follow-up; SD: Standard Deviation; T: time; *statistically significant for *p* < 0.05. Note: p-values are reported in bold, while effect size in plain character

No significant association was found between LC and over-time variation of hippocampal volume or MMSE (Supplem. Tables 3 and 44).

## Discussion

Profiting of LC-MRI, we were able to evaluate the time-dependent degeneration of LC occurring in patients belonging to the AD clinical continuum. Even though this phenomenon has been already demonstrated in neuropathological studies (Braak et al. 2011; Theofilas et al. 2017), only a few neuroimaging longitudinal ones have been performed on LC (Jacobs et al. 2023; Dahl et al. 2023). In 2023, Jacobs and colleagues explored the variation of LC over time in a population of autosomal dominant AD cases. They found that the decrease in LC signal was associated with tau accumulation in the precuneus, assessed through PET (Jacobs et al. 2023). In the same year, Dahl and colleagues found that in cognitively intact elderly individuals, the reduction in LC signal is associated with worsening memory performance (Dahl et al. 2023), aligning with previous cross-sectional LC-MRI studies (Dahl et al. 2019; Liu et al. 2020a).

It is worth noting that our study is the first LC-MRI follow-up analysis performed in late-onset AD, and it parallels the neuropathology findings reported by Theofilas and colleagues in 2017 (Theofilas et al. 2017). In that study, the authors investigated the degeneration of the LC across neurofibrillary pathology-related Braak stages (BB stage-Braak et al. 2011) in a cohort of AD patients and aged controls, using stereological analysis to precisely estimate LC neuronal number and thus providing very reliable data concerning the involvement of LC during both physiological and pathological ageing (Theofilas et al. 2017). Theofilas and colleagues reported progressive LC neuron loss and atrophy along BB stages, from I to VI (Theofilas et al. 2017). Moreover, they found that LC cell number reduction becomes statistically significant in BB mid-stages (III-IV), with the rate of cellular death becoming dramatic in advanced stages (V-VI) (Theofilas et al. 2017). Interestingly, our radiological findings match quite well the neuropathological ones just quoted, as we observed a global decrease of the LC_CR_ parameter, which can be considered as a proxy of LC neuronal density (Keren et al. 2015). This was shown considering both the whole population and each diagnostic group separately, since we observed a significant LC_CR_ reduction not only in MCI individuals, but also in ADD patients which are well known to be affected by more advanced AD pathology stages (Thal et al. 2002; Braak et al. 2011). Furthermore, Theofilas et al. showed that LC volumetric shrinkage and atrophy are related to BB staging as well, and follow a progressive reduction pattern, which is particularly evident in the brain of subjects affected by I-to-IV BB stages (Theofilas et al. 2017). Even in this case, our results are in line with this *post-mortem* evidence, since we found a significant decrease in values of the volumetric parameter LC_VOX_ in MCI individuals, while in ADD patients we only observed a trend, which, however, did not survive the multiple comparison correction. These data may suggest that LC-MRI enables the estimation of progressive LC atrophy, with a trend slope that could be steeper in the initial stages of the disease compared to what occurring in demented patients.

Finally, Grinberg’s group study showed that LC does not suffer from a physiological and age-dependent alteration, but, rather, its degeneration is strictly related to the occurrence of AD pathology (Theofilas et al. 2017). If, on the one hand, their observation is crucial for hypothesizing a specific role of LC impairment in AD pathogenesis, on the other it highlights the most important of the limitations of the present study, which is represented by the lack of radiological follow-up data on cognitively intact healthy controls. In line with this, we could not possibly draw any inference on LC-MRI features during healthy aging. However, it should be noted that such a limitation does not hamper the analysis we performed in patients, which clearly shows a time-dependent LC degeneration and is based on baseline MRI assessments in which LC loss of integrity was already present (Galgani et al. 2023). Moreover, the available literature might allow to speculate on what might be observed in cognitively-spared ageing. Cross-sectional studies reported that in the absence of cognitive decline, LC-MRI signal does not vary among different age groups (Giorgi et al. 2021; Al Haddad et al. 2023). Other studies revealed an age-dependent reduction of the signal in the rostral part of the LC, which however was inevitably related to a decline in the memory performances and in global cognition (Liu et al. 2019, 2020a; Dahl et al. 2019). The above-cited recent longitudinal study (Dahl et al. 2023) showed that reduction of LC signal during follow-up was associated with an increased risk of developing memory impairment (Dahl et al. 2023). Altogether, these pieces of evidence might suggest that LC-MRI signal may not undergo any kind of alteration in neurodegenerative-free ageing, as also shown by neuropathological evidence (Theofilas et al. 2017), while its decrease might be the sign of an ongoing degenerative phenomenon.

The second limitation of our study is the lack of concomitant in vivo neuroimaging information on the BB stages of patients included (e.g., Tau protein tracers PET) and of amyloid biomarkers. This reduced the accuracy of the evaluation of AD pathology in our sample, preventing us from linearly comparing our results with neuropathological ones and from excluding the occurrence of AD-mimicking pathology, such as primary age-related tauopathy (PART) (Crary et al. 2014) or limbic-predominant age-related TDP-43 encephalopathy (LATE) (Nelson et al. 2019; Liu et al. 2020b). Concerning PART, *post-mortem* data seems to indicate a common path with AD, with a similar involvement of LC by tau pathology (Kaufman et al. 2018; Zhu et al. 2019). For LATE, as far as we know, no neuropathological studies on the involvement of the LC have been performed yet and thus we cannot rule out its possible influence. To account for this limitation, we provided a thorough clinical, neuropsychological, and neuroradiological characterization of the study population (Galgani et al. 2023), and we believe that this might represent a solid background for discussing and interpreting our results.

Furthermore, we chose to include only patients affected by typical AD or amnestic MCI individuals. These subjects are more likely to bear AD pathology, with a predominant involvement of the limbic structures (Braak and Braak 1991). As the link of AD with LC degeneration has been defined by several studies (Dahl et al. 2019; Van Egroo et al. 2023; Galgani et al. 2023; Bell et al. 2023), this allowed us to frame our data and their interpretation in a well-explored context from a neuroanatomical and pathological point of view. In atypical forms of AD or non-amnestic MCI subjects, drawing similar assumption would have been more challenging. To the best of our knowledge, only one LC-MRI study has been performed up to now in patients with atypical AD (Olivieri et al. 2019). Even though those authors found that the LC degeneration occurs to a similar extent both in typical and atypical AD (Olivieri et al. 2019), the specific reasons for which AD pathology might affect the LC projections to either frontotemporal or occipitoparietal cortices rather than the limbic ones have not been elucidated yet.

The last limitation we want to acknowledge is the low number of patients involved (*N* = 57); even though this was sufficient to allow us to disclose the effect of time on LC degeneration, it probably hindered the accurate assessment of the effects of other factors, e.g. the baseline diagnosis.

## Conclusions

In conclusion, we here report the in vivo evidence of LC degeneration during AD progression. Even though this piece of data could be already foreseen from neuropathological studies, it is the first time, as far as we know, that the occurrence of this phenomenon has been demonstrated in vivo in patients, profiting from a longitudinal LC-MRI assessment. These results not only support the hypothesis of the involvement of LC in AD pathology, but also strengthen the reliability of LC-MRI as a tool useful to assess LC features in physiological and pathological conditions. Indeed, this study adds to the list of LC-MRI studies that have repeatedly confirmed experimental and *post-mortem* data (Beardmore et al. 2021). Further studies are warranted not only to confirm our findings, but also to better explore the role of LC integrity in cognitively intact elderly subjects. For instance, if a longitudinal radiological study, similar to the current one, were conducted on a large cohort of older adults stratified based on AD-related biomarkers and followed clinically over an extended period of time to monitor the occurrence of cognitive impairment, it could reveal whether an early alteration of LC triggers the onset of AD, as suggested by experimental studies (Weinshenker 2018). This might also pave the way to NA-targeted therapy as an early disease-modifying treatment of AD.

### Electronic supplementary material

Below is the link to the electronic supplementary material.

Supplementary Material 1

Supplementary Material 2

Supplementary Material 3

Supplementary Material 4

Supplementary Material 5

Supplementary Material 6

Supplementary Material 7

Supplementary Material 8

## Data Availability

The dataset generated during the current study is available from the corresponding author on reasonable request.
