# Supplementary material for: The degeneration of locus coeruleus occurring during Alzheimer’s disease clinical progression: a neuroimaging follow-up investigation
Source: Brain Struct Funct. 2024 Apr 16;229(5):1317–25. doi: 10.1007/s00429-024-02797-1 (PMC11147916; doi:10.1007/s00429-024-02797-1)
Supplement: Supplementary file 1 — Supplementary Material 1 [file 429_2024_2797_MOESM1_ESM.pdf]

Supplementary Table 5. Wilcoxon paired-sample tests between rostral and caudal half of the LC.

			All subjects			ncMCI			cMCI			ADD		
			Caudal half	Rostral half	p	Caudal half	Rostral half	p	Caudal half	Rostral half	p	Caudal half	Rostral half	p
T0	LC_{CR}	M	0.043	0.036	0.076	0.051	0.042	0.228	0.039	0.033	0.376	0.033	0.029	0.480
		SD	0.036	0.027		0.037	0.029		0.035	0.026		0.033	0.021	
	LC_{VOX}	M	22.110	18.390	0.114	25.580	22.310	0.231	20.680	16.530	0.150	16.830	12.830	0.398
		SD	18.147	16.850		18.424	19.066		18.806	16.067		16.247	10.994	
T1	LC_{CR}	M	0.002	0.000	0.538	0.011	0.010	0.751	0.001	-0.007	0.126	-0.015	-0.009	0.433
		SD	0.030	0.025		0.030	0.025		0.025	0.020		0.029	0.025	
	LC_{VOX}	M	11.351	8.298	0.198	16.308	12.423	0.073	8.947	4.842	0.204	4.417	4.833	0.610
		SD	11.696	10.959		11.723	14.269		11.659	5.429		6.417	5.149	
Absolute variation	LC_{CR}	M	-0.041	-0.036	0.071	-0.040	-0.032	0.276	-0.038	-0.040	0.748	-0.048	-0.038	0.272
		SD	0.027	0.027		0.022	0.020		0.023	0.026		0.039	0.039	
	LC_{VOX}	M	-10.754	-10.088	0.578	-9.269	-9.885	0.819	-11.737	-11.684	0.810	-12.417	-8.000	0.213
		SD	13.410	14.541		13.346	14.831		11.855	16.327		16.456	11.449	

Legend to tables. Absolute values of LC-MRI parameters for the rostral and caudal half of LC (mean and standard deviation) are reported, together with the variation measured between T1 and T0 (T1-T0). ADD: Alzheimer's Disease Dementia; T0: Baseline; T1: End of the follow-up; M: Mean; MCI: Mild Cognitive Impairment; cMCI: MCI converter; ncMCI: MCI non-converter; p: adjusted p-value for FDR multiple comparison correction; SD: Standard Deviation; *statistically significant for $p < 0.05$.

From the paper "The degeneration of Locus Coeruleus occurring during Alzheimer's Disease clinical progression: a neuroimaging follow-up investigation" published on "Brain Structure and Function" by Alessandro Galgani. Francesco Lombardo. Francesca Frija. Nicola Martini. Gloria Tognoni. Nicola Pavese and Filippo S. Giorgi. (*Corresponding author: Department of Translational Research and of New Surgical and Medical Technologies. University of Pisa. e-mail address: filippo.giorgi@unipi.it).*
