# Supplementary material for: The degeneration of locus coeruleus occurring during Alzheimer’s disease clinical progression: a neuroimaging follow-up investigation
Source: Brain Struct Funct. 2024 Apr 16;229(5):1317–25. doi: 10.1007/s00429-024-02797-1 (PMC11147916; doi:10.1007/s00429-024-02797-1)
Supplement: Supplementary file 2 — Supplementary Material 2 [file 429_2024_2797_MOESM2_ESM.pdf]

Supplementary Table 6. Wilcoxon paired-sample tests between left and right LC.

			All subjects			ncMCI			cMCI			ADD		
			**Right**	**Left**	**p**	**Right**	**Left**	**p**	**Right**	**Left**	**p**	**Right**	**Left**	**p**
**T0**	LC_{CR}	M	0.032	0.046	**0.001**	0.036	0.055	**0.001**	0.031	0.039	0.159	0.025	0.037	0.071
		SD	0.028	0.033		0.031	0.032		0.026	0.034		0.023	0.030	
	LC_{VOX}	M	14.930	22.158	**0.001**	16.346	27.346	**0.000**	15.053	19.053	0.338	11.667	15.833	0.266
		SD	14.413	18.092		15.955	18.417		15.054	18.422		9.585	14.702	
**T1**	LC_{CR}	M	-0.008	0.008	**0.000**	0.000	0.018	**0.000**	-0.011	0.002	**0.048**	-0.024	-0.001	0.090
		SD	0.026	0.025		0.026	0.025		0.022	0.023		0.027	0.024	
	LC_{VOX}	M	6.820	11.110	**0.000**	9.580	16.270	**0.004**	6.000	6.890	0.422	2.170	6.580	0.108
		SD	9.134	10.720		10.534	11.681		8.253	8.640		4.282	6.067	
**Absolute variation**	LC_{CR}	M	-0.040	-0.038	0.355	-0.036	-0.038	0.790	-0.042	-0.037	0.573	-0.049	-0.038	0.084
		SD	0.025	0.031		0.023	0.024		0.022	0.034		0.035	0.040	
	LC_{VOX}	M	-8.105	-11.053	0.232	-6.769	-11.077	0.274	-9.053	-12.158	0.368	-9.500	-9.250	0.637
		SD	12.134	14.425		12.011	14.908		13.612	15.145		10.518	13.164	

Legend to tables. Absolute values of LC-MRI parameters for the left and right LCs (mean and standard deviation) are reported, together with the variation measured between T1 and T0 (T1-T0). ADD: Alzheimer's Disease Dementia; T0: Baseline; T1: End of the follow-up; M: Mean; MCI: Mild Cognitive Impairment; cMCI: MCI converter; ncMCI: MCI non-converter; p: adjusted p-value for FDR multiple comparison correction; SD: Standard Deviation; *statistically significant for $p < 0.05$.

From the paper "The degeneration of Locus Coeruleus occurring during Alzheimer's Disease clinical progression: a neuroimaging follow-up investigation" published on "Brain Structure and Function" by Alessandro Galgani. Francesco Lombardo. Francesca Frija. Nicola Martini. Gloria Tognoni. Nicola Pavese and Filippo S. Giorgi. (*Corresponding author: Department of Translational Research and of New Surgical and Medical Technologies. University of Pisa. e-mail address: filippo.giorgi@unipi.it).*
