# Supplementary material for: The degeneration of locus coeruleus occurring during Alzheimer’s disease clinical progression: a neuroimaging follow-up investigation
Source: Brain Struct Funct. 2024 Apr 16;229(5):1317–25. doi: 10.1007/s00429-024-02797-1 (PMC11147916; doi:10.1007/s00429-024-02797-1)
Supplement: Supplementary file 3 — Supplementary Material 3 [file 429_2024_2797_MOESM3_ESM.pdf]

Supplementary Table 4. Spearman's correlation test for LC and MMSE

Correlation between LC-MRI parameters and MMSE scores both at baseline and at follow-up

				MMSE	
				**Bas**	**FU**
**Baseline**	**LC_{CR}**	LC complex	Rho	0.243	0.223
			p-value	0.071	0.095
		LC complex - Lower half	Rho	0.106	0.178
			p-value	0.435	0.186
		LC complex - Upper half	Rho	**0.350**	0.193
			p-value	0.004*	0.150
		Right LC	Rho	0.133	0.099
			p-value	0.329	0.465
		Left LC	Rho	0.287	0.226
			p-value	0.100	0.091
	**LC_{VOX}**	LC complex	Rho	0.211	0.202
			p-value	0.119	0.131
		LC complex - Lower half	Rho	0.055	0.171
			p-value	0.687	0.203
		LC complex - Upper half	Rho	**0.381**	0.193
			p-value	0.040*	0.149
		Right LC	Rho	0.137	0.090
			p-value	0.313	0.505
		Left LC	Rho	0.221	0.228
			p-value	0.101	0.088
**Follow-up**	**LC_{CR}**	LC complex	Rho	0.293	**0.423**
			p-value	0.093	0.003*
		LC complex - Lower half	Rho	0.212	**0.363**
			p-value	0.117	0.009*
		LC complex - Upper half	Rho	0.300	**0.366**
			p-value	0.120	0.008*
		Right LC	Rho	0.217	**0.345**
			p-value	0.109	0.011*
		Left LC	Rho	0.317	**0.403**
			p-value	0.170	0.004*
	**LC_{VOX}**	LC complex	Rho	0.219	**0.471**
			p-value	0.105	0.001*
		LC complex - Lower half	Rho	0.253	**0.478**
			p-value	0.060	0.001*
		LC complex - Upper half	Rho	0.173	**0.325**
			p-value	0.203	0.014*
		Right LC	Rho	0.159	**0.287**
			p-value	0.242	0.030*
		Left LC	Rho	0.243	**0.484**
			p-value	0.071	0.001*

Correlation between overtime variation of LC-MRI parameters and MMSE scores

				MMSE variation
LC variation	LC_{CR}	LC complex	Rho	0.210
			p-value	0.121
		LC complex - Lower half	Rho	0.204
			p-value	0.132
		LC complex - Upper half	Rho	0.186
			p-value	0.170
		Right LC	Rho	0.216
			p-value	0.109
		Left LC	Rho	0.180
			p-value	0.185
	LC_{VOX}	LC complex	Rho	0.242
			p-value	0.072
		LC complex - Lower half	Rho	0.148
			p-value	0.275
		LC complex - Upper half	Rho	0.172
			p-value	0.204
		Right LC	Rho	0.133
			p-value	0.329
		Left LC	Rho	0.145
			p-value	0.287

Legend to tables. The variation of both LC-MRI parameters and MMSE scores were calculated as the difference between follow-up and baseline assessments. Bas: Baseline; FU: Follow-up; All reported p-values were adjusted for FDR multiple comparison correction; *statistically significant for $p < 0.05$.

From the paper "The degeneration of Locus Coeruleus occurring during Alzheimer's Disease clinical progression: a neuroimaging follow-up investigation" published on "Brain Structure and Function" by Alessandro Galgani. Francesco Lombardo. Francesca Frija. Nicola Martini. Gloria Tognoni. Nicola Pavese and Filippo S. Giorgi. (*Corresponding author: Department of Translational Research and of New Surgical and Medical Technologies. University of Pisa. e-mail address: filippo.giorgi@unipi.it).*
