# Supplementary material for: The degeneration of locus coeruleus occurring during Alzheimer’s disease clinical progression: a neuroimaging follow-up investigation
Source: Brain Struct Funct. 2024 Apr 16;229(5):1317–25. doi: 10.1007/s00429-024-02797-1 (PMC11147916; doi:10.1007/s00429-024-02797-1)
Supplement: Supplementary file 4 — Supplementary Material 4 [file 429_2024_2797_MOESM4_ESM.pdf]

Supplementary Table 3. Spearman's correlation test for LC and hippocampal volume

				Hippocampal volume							
				Baseline		Follow-up		Absolute variation		Relative variation	
				Right	Left	Right	Left	Right	Left	Right	Left
**Baseline**	**LC_{CR}**	LC complex	Rho	0.053	0.010	-0.008	0.025	-0.029	-0.026	-0.038	-0.010
			p-value	0.697	0.940	0.950	0.856	0.832	0.849	0.782	0.943
		LC complex - Lower half	Rho	0.059	0.035	0.042	0.048	-0.004	-0.033	-0.012	-0.024
			p-value	0.663	0.795	0.756	0.723	0.976	0.809	0.931	0.861
		LC complex - Upper half	Rho	0.005	-0.043	-0.032	0.009	-0.010	0.010	-0.018	0.029
			p-value	0.971	0.753	0.811	0.948	0.944	0.940	0.893	0.829
		Right LC	Rho	0.080	0.103	0.034	0.130	-0.017	-0.003	-0.017	0.017
			p-value	0.556	0.444	0.802	0.334	0.901	0.980	0.898	0.900
		Left LC	Rho	0.046	-0.051	-0.026	-0.042	-0.032	-0.028	-0.047	-0.024
			p-value	0.737	0.709	0.847	0.759	0.810	0.838	0.726	0.859
	**LC_{VOX}**	LC complex	Rho	0.008	0.039	-0.042	0.029	-0.025	-0.041	-0.030	-0.021
			p-value	0.954	0.771	0.756	0.829	0.855	0.759	0.823	0.877
		LC complex - Lower half	Rho	0.006	0.027	-0.031	0.017	-0.021	-0.052	-0.030	-0.039
			p-value	0.963	0.845	0.820	0.902	0.876	0.702	0.824	0.773
		LC complex - Upper half	Rho	0.082	0.110	-0.005	0.063	-0.061	-0.054	-0.064	-0.020
			p-value	0.544	0.416	0.968	0.642	0.654	0.691	0.636	0.881
		Right LC	Rho	0.018	0.143	-0.004	0.148	-0.005	0.008	-0.005	0.031
			p-value	0.893	0.287	0.976	0.273	0.973	0.955	0.973	0.817
		Left LC	Rho	0.024	-0.025	-0.017	-0.034	-0.016	-0.047	-0.030	-0.031
			p-value	0.861	0.853	0.900	0.803	0.903	0.728	0.824	0.816
**Follow-up**	**LC_{CR}**	LC complex	Rho	0.062	0.115	0.274	0.341	0.171	0.217	0.170	0.235
			p-value	0.646	0.392	0.156	0.072	0.204	0.105	0.207	0.078
		LC complex - Lower half	Rho	0.042	0.055	0.204	0.234	0.089	0.149	0.082	0.158
			p-value	0.755	0.686	0.128	0.080	0.509	0.269	0.546	0.241
		LC complex - Upper half	Rho	0.018	0.098	0.225	0.279	0.239	0.184	0.239	0.202
			p-value	0.896	0.470	0.092	0.288	0.073	0.170	0.074	0.131
		Right LC	Rho	-0.023	0.068	0.219	0.274	0.241	0.250	0.230	0.267
			p-value	0.867	0.615	0.101	0.312	0.071	0.061	0.086	0.180
		Left LC	Rho	0.021	0.075	0.226	0.275	0.159	0.224	0.151	0.234
			p-value	0.875	0.582	0.091	0.304	0.237	0.095	0.262	0.080

	LCVOX	LC complex	Rho	-0.072	0.065	0.189	0.244	0.327	0.215	0.322	0.240
			p-value	0.593	0.633	0.158	0.068	0.104	0.109	0.060	0.072
		LC complex - Lower half	Rho	0.058	0.042	0.260	0.224	0.193	0.166	0.181	0.178
			p-value	0.666	0.758	0.050	0.094	0.150	0.218	0.178	0.186
		LC complex - Upper half	Rho	0.058	0.169	0.189	0.331	0.088	0.162	0.095	0.182
			p-value	0.669	0.210	0.159	0.096	0.517	0.227	0.482	0.176
		Right LC	Rho	-0.074	-0.059	0.140	0.115	0.239	0.203	0.214	0.202
			p-value	0.584	0.665	0.299	0.395	0.073	0.131	0.109	0.132
		Left LC	Rho	0.027	0.130	0.225	0.326	0.183	0.222	0.182	0.248
			p-value	0.844	0.333	0.092	0.104	0.172	0.097	0.175	0.063
**LC variation**	**LC_{CR}**	LC complex	Rho	0.005	0.077	0.206	0.253	0.143	0.223	0.157	0.219
			p-value	0.969	0.568	0.125	0.058	0.288	0.095	0.243	0.101
		LC complex - Lower half	Rho	0.018	0.033	0.131	0.197	0.027	0.194	0.042	0.194
			p-value	0.893	0.809	0.333	0.142	0.844	0.148	0.758	0.149
		LC complex - Upper half	Rho	0.061	0.124	0.262	0.232	0.213	0.151	0.221	0.143
			p-value	0.653	0.357	0.392	0.083	0.111	0.263	0.098	0.289
		Right LC	Rho	-0.028	-0.081	0.176	0.028	0.176	0.123	0.168	0.102
			p-value	0.837	0.549	0.189	0.838	0.190	0.361	0.211	0.452
		Left LC	Rho	0.108	0.188	0.180	0.302	0.030	0.175	0.055	0.182
			p-value	0.424	0.161	0.180	0.176	0.823	0.194	0.684	0.176
	**LC_{VOX}**	LC complex	Rho	-0.076	-0.047	0.193	0.160	0.231	0.254	0.236	0.230
			p-value	0.573	0.728	0.151	0.235	0.083	0.057	0.077	0.085
		LC complex - Lower half	Rho	0.018	0.011	0.196	0.187	0.121	0.234	0.130	0.219
			p-value	0.894	0.935	0.144	0.165	0.368	0.079	0.334	0.102
		LC complex - Upper half	Rho	-0.107	-0.050	0.108	0.087	0.220	0.155	0.223	0.131
			p-value	0.427	0.714	0.423	0.521	0.099	0.251	0.096	0.332
		Right LC	Rho	-0.079	-0.187	0.074	-0.085	0.147	0.121	0.135	0.087
			p-value	0.560	0.163	0.584	0.528	0.276	0.370	0.317	0.521
		Left LC	Rho	0.017	0.103	0.213	0.253	0.165	0.206	0.183	0.195
			p-value	0.901	0.445	0.112	0.058	0.219	0.125	0.173	0.146

Legend to tables. The variation of both LC-MRI parameters and hippocampal volume (absolute variation) were calculated as the difference between follow-up and baseline assessments. For the hippocampal volume the relative variation was also extrapolated, which is the ratio between the absolute variation and the baseline volume. Hippocampal volume was standardized for TIV. Bas: Baseline; FU: Follow-up; All reported p-values were adjusted for FDR multiple comparison correction; *statistically significant for $p < 0.05$.

From the paper "The degeneration of Locus Coeruleus occurring during Alzheimer's Disease clinical progression: a neuroimaging follow-up investigation" published on "Brain Structure and Function" by Alessandro Galgani. Francesco Lombardo. Francesca Frija. Nicola Martini. Gloria Tognoni. Nicola Pavese and Filippo S. Giorgi. (*Corresponding author: Department of Translational Research and of New Surgical and Medical Technologies. University of Pisa. e-mail address: filippo.giorgi@unipi.it).*
