# Supplementary material for: The degeneration of locus coeruleus occurring during Alzheimer’s disease clinical progression: a neuroimaging follow-up investigation
Source: Brain Struct Funct. 2024 Apr 16;229(5):1317–25. doi: 10.1007/s00429-024-02797-1 (PMC11147916; doi:10.1007/s00429-024-02797-1)
Supplement: Supplementary file 5 — Supplementary Material 5 [file 429_2024_2797_MOESM5_ESM.pdf]

Supplementary Table 1. Hippocampal volume analysis

Hippocampal volume variation over time – Wilcoxon paired test

	T	All subjects			ncMCI			cMCI			ADD		
		M	SD	p	M	SD	p	M	SD	p	M	SD	p
**Hippocampus**													
Left	Bas	0.472	0.060	**0.003***	0.483	0.054	**0.304**	0.455	0.072	**0.027***	0.476	0.050	**0.099**
	FU	0.445	0.066		0.475	0.049		0.413	0.074		0.429	0.061	
Right	Bas	0.462	0.066	**0.015***	0.470	0.061	**0.889**	0.456	0.066	**0.011***	0.457	0.077	**0.060**
	FU	0.440	0.068		0.474	0.051		0.410	0.069		0.415	0.060	

Hippocampal volume difference among groups – Kruskal Wallis test and Mann-Whitney post-hoc

Hippocampus	T	KW	Mann-Whitney p-value		
		p-value	**cMCI vs ADD**	**cMCI vs ncMCI**	**ADD vs ncMCI**
Left	Bas	0.387			
	FU	0.004*	0.497	0.001*	0.042*
Right	Bas	0.679			
	FU	0.002*	0.959	0.002*	0.007*

Legend to tables. ADD: Alzheimer's Disease Dementia; Bas: Baseline; ES: Effect size; FU: Follow-up; M: Mean; MCI: Mild Cognitive Impairment; cMCI: MCI converter; ncMCI: MCI non-converter; SD: Standard Deviation; T: time; *statistically significant for $p < 0.05$.

From the paper "The degeneration of Locus Coeruleus occurring during Alzheimer's Disease clinical progression: a neuroimaging follow-up investigation" published on "Brain Structure and Function" by Alessandro Galgani. Francesco Lombardo. Francesca Frija. Nicola Martini. Gloria Tognoni. Nicola Pavese and Filippo S. Giorgi. (*Corresponding author: Department of Translational Research and of New Surgical and Medical Technologies. University of Pisa. e-mail address: filippo.giorgi@unipi.it).*
