# Supplementary material for: The degeneration of locus coeruleus occurring during Alzheimer’s disease clinical progression: a neuroimaging follow-up investigation
Source: Brain Struct Funct. 2024 Apr 16;229(5):1317–25. doi: 10.1007/s00429-024-02797-1 (PMC11147916; doi:10.1007/s00429-024-02797-1)
Supplement: Supplementary file 6 — Supplementary Material 6 [file 429_2024_2797_MOESM6_ESM.pdf]

Supplementary table 2. Linear Mixed Models assessing the effect of time and diagnosis on LC-MRI parameters variation.

LC COMPLEX							
**LC_{CR} – Test of fixed effects**							
Source	Num df	Den df	F	p-value			
Intercept	1	54	0.511	0.478			
Time	1	54	140.07	<0.001*			
Diagnosis	2	54	3.19	0.049*			
Time*Diagnosis	2	54	0.289	0.750			
**Pairwise Comparisons**							
		Mean Difference	Std. Error	df	Sig.	95% Confidence Interval	
						Lower Bound	Upper Bound
Baseline	Follow-up	1.221	0.103	54	<0.001*	1.014	1.428
Follow-up	Baseline	-1.221	0.103	54	<0.001*	-1.428	-1.014
ncMCI	cMCI	0.368	0.208	54	0.082	-0.048	0.785
	ADD	0.558	0.240	54	0.024*	0.076	1.039
cMCI	ncMCI	-0.368	0.208	54	0.082	-0.785	0.048
	ADD	0.189	0.254	54	0.459	-0.319	0.698
ADD	ncMCI	-0.558	0.240	54	0.024*	-1.039	-0.076
	cMCI	-0.189	0.254	54	0.459	-0.698	0.319
**LC_{VOX} – Test of fixed effects**							
Source	Num df	Den df	F	p-value			
Intercept	1	54	0.493	0.486			
Time	1	54	37.623	<0.001*			
Diagnosis	2	54	3.123	0.052			
Time*Diagnosis	2	54	0.124	0.883			
**Pairwise Comparisons**							
		Mean Difference	Std. Error	df	Sig.	95% Confidence Interval	
						Lower Bound	Upper Bound
Baseline	Follow-up	0.738	0.120	54	<0.001*	0.497	0.979
Follow-up	Baseline	-0.738	0.120	54	<0.001*	-0.979	-0.497

LC – CAUDAL PART							
**LC_{CR} – Test of fixed effects**							
Source	Num df	Den df	F	p-value			
Intercept	1	54	0.425	0.517			
Time	1	54	127.496	<0.001*			
Diagnosis	2	54	2.380	0.102			
Time*Diagnosis	2	54	0.534	0.589			
**Pairwise Comparisons**							
		Mean Difference	Std. Error	df	Sig.	95% Confidence Interval	
						Lower Bound	Upper Bound
Baseline	Follow-up	1.089	0.096	54	<0.001*	0.895	1.282
Follow-up	Baseline	-1.089	0.096	54	<0.001*	-1.282	-0.895
**LC_{VOX} – Test of fixed effects**							
Source	Num df	Den df	F	p-value			
Intercept	1	54	0.369	0.546			
Time	1	54	30.643	<0.001*			
Diagnosis	2	54	2.551	0.087			
Time*Diagnosis	2	54	0.205	0.815			
**Pairwise Comparisons**							
		Mean Difference	Std. Error	df	Sig.	95% Confidence Interval	
						Lower Bound	Upper Bound
Baseline	Follow-up	0.830	0.150	54	<0.001*	0.530	1.131
Follow-up	Baseline	-0.830	0.150	54	<0.001*	-1.131	-0.530

LC – ROSTRAL PART							
**LC_{CR} – Test of fixed effects**							
Source	Num df	Den df	F	p-value			
Intercept	1	54	0.449	0.505			
Time	1	54	95.888	<0.001*			
Diagnosis	2	54	3.346	0.043			
Time*Diagnosis	2	54	0.484	0.619			
**Pairwise Comparisons**							
		Mean Difference	Std. Error	df	Sig.	95% Confidence Interval	
						Lower Bound	Upper Bound
Baseline	Follow-up	1.165	0.119	54	<0.001*	0.927	1.404
Follow-up	Baseline	-1.165	0.119	54	<0.001*	-1.404	-0.927
ncMCI	cMCI	0.428	0.204	54	0.041*	0.018	0.838
	ADD	0.514	0.236	54	0.034*	0.041	0.988
cMCI	ncMCI	-0.428	0.204	54	0.041*	-0.838	-0.018
	ADD	0.086	0.250	54	0.731	-0.414	0.587
ADD	ncMCI	-0.514	0.236	54	0.034*	-0.988	-0.041
	cMCI	-0.086	0.250	54	0.731	-0.587	0.414
**LC_{VOX} – Test of fixed effects**							
Source	Num df	Den df	F	p-value			
Intercept	1	54	0.635	0.429			
Time	1	54	11.897	0.001*			
Diagnosis	2	54	3.813	0.028*			
Time*Diagnosis	2	54	0.121	0.886			
**Pairwise Comparisons**							
		Mean Difference	Std. Error	df	Sig.	95% Confidence Interval	
						Lower Bound	Upper Bound
Baseline	Follow-up	0.493	0.143	54	0.001*	0.207	0.780
Follow-up	Baseline	-0.493	0.143	54	0.001*	-0.780	-0.207
ncMCI	cMCI	0.442	0.240	54	0.071	-0.039	0.923
	ADD	0.719	0.278	54	0.012*	0.162	1.275
cMCI	ncMCI	-0.442	0.240	54	0.071	-0.923	0.039
	ADD	0.277	0.293	54	0.350	-0.312	0.865
ADD	ncMCI	-0.719	0.278	54	0.012*	-1.275	-0.162
	cMCI	-0.277	0.293	54	0.350	-0.865	0.312

RIGHT LC							
**LC_{CR} – Test of fixed effects**							
Source	Num df	Den df	F	p-value			
Intercept	1	54	0.450	0.505			
Time	1	54	142.592	<0.001*			
Diagnosis	2	54	2.449	0.096			
Time*Diagnosis	2	54	1.045	0.359			
**Pairwise Comparisons**							
		Mean Difference	Std. Error	df	Sig.	95% Confidence Interval	
						Lower Bound	Upper Bound
Baseline	Follow-up	1.248	0.105	54	<0.001*	1.039	1.458
Follow-up	Baseline	-1.248	0.105	54	<0.001*	-1.458	-1.039
**LC_{VOX} – Test of fixed effects**							
Source	Num df	Den df	F	p-value			
Intercept	1	54	0.265	0.609			
Time	1	54	24.331	<0.001*			
Diagnosis	2	54	1.416	0.252			
Time*Diagnosis	2	54	0.287	0.751			
**Pairwise Comparisons**							
		Mean Difference	Std. Error	df	Sig.	95% Confidence Interval	
						Lower Bound	Upper Bound
Baseline	Follow-up	0.665	0.135	54	<0.001*	0.395	0.936
Follow-up	Baseline	-0.665	0.135	54	<0.001*	-0.936	-0.395

LEFT LC							
**LC_{CR} – Test of fixed effects**							
Source	Num df	Den df	F	p-value			
Intercept	1	54	0.474	0.494			
Time	1	54	74.352	<0.001*			
Diagnosis	2	54	3.613	0.034			
Time*Diagnosis	2	54	0.003	0.997			
**Pairwise Comparisons**							
		Mean Difference	Std. Error	df	Sig.	95% Confidence Interval	
						Lower Bound	Upper Bound
Baseline	Follow-up	1.079	0.125	54	<0.001*	0.828	1.330
Follow-up	Baseline	-1.079	0.125	54	<0.001*	-1.330	-0.828
ncMCI	cMCI	0.458	0.208	54	0.032*	0.042	0.874
	ADD	0.537	0.240	54	0.029*	0.056	1.019
cMCI	ncMCI	-0.458	0.208	54	0.032*	-0.874	-0.042
	ADD	0.079	0.254	54	0.756	-0.429	0.588
ADD	ncMCI	-0.537	0.240	54	0.029*	-1.019	-0.056
	cMCI	-0.079	0.254	54	0.756	-0.588	0.429
**LC_{VOX} – Test of fixed effects**							
Source	Num df	Den df	F	p-value			
Intercept	1	54	0.578	0.450			
Time	1	54	28.186	<0.001*			
Diagnosis	2	54	4.311	0.018*			
Time*Diagnosis	2	54	0.145	0.865			
**Pairwise Comparisons**							
		Mean Difference	Std. Error	df	Sig.	95% Confidence Interval	
						Lower Bound	Upper Bound
Baseline	Follow-up	0.685	0.129	54	<0.001*	0.426	0.944
Follow-up	Baseline	-0.685	0.129	54	<0.001*	-0.944	-0.426
ncMCI	cMCI	0.559	0.235	54	0.021*	0.088	1.029
	ADD	0.670	0.271	54	0.017*	0.126	1.215
cMCI	ncMCI	-0.559	0.235	54	0.021*	-1.029	-0.088
	ADD	0.112	0.287	54	0.699	-0.463	0.687
ADD	ncMCI	-0.670	0.271	54	0.017*	-1.215	-0.126
	cMCI	-0.112	0.287	54	0.699	-0.687	0.463

Please. note that the values of LC-MRI parameters were z-standardized to perform this set of analysis. Redundant and non-significant results were not reported. *statistically significant for $p < 0.05$.

Legend to tables. ADD: Alzheimer's Disease Dementia; MCI: Mild Cognitive Impairment; cMCI: MCI converter; ncMCI: MCI non-converter.

From the paper "The degeneration of Locus Coeruleus occurring during Alzheimer's Disease clinical progression: a neuroimaging follow-up investigation" published on "Brain Structure and Function" by Alessandro Galgani. Francesco Lombardo. Francesca Frija. Nicola Martini. Gloria Tognoni. Nicola Pavese and Filippo S. Giorgi. (*Corresponding author: Department of Translational Research and of New Surgical and Medical Technologies. University of Pisa. e-mail address: filippo.giorgi@unipi.it).*
