# Supplementary material for: The degeneration of locus coeruleus occurring during Alzheimer’s disease clinical progression: a neuroimaging follow-up investigation
Source: Brain Struct Funct. 2024 Apr 16;229(5):1317–25. doi: 10.1007/s00429-024-02797-1 (PMC11147916; doi:10.1007/s00429-024-02797-1)
Supplement: Supplementary file 7 — Supplementary Material 7 [file 429_2024_2797_MOESM7_ESM.pdf]

Supplementary Methods & Results S1

From the paper "The degeneration of Locus Coeruleus occurring during Alzheimer's Disease clinical progression: a neuroimaging follow-up investigation" published on "Brain Structure and Function" by Alessandro Galgani. Francesco Lombardo. Francesca Frija. Nicola Martini. Gloria Tognoni. Nicola Pavese and Filippo S. Giorgi. (*Corresponding author: Department of Translational Research and of New Surgical and Medical Technologies. University of Pisa. e-mail address: filippo.giorgi@unipi.it).*

Supplementary Methods

Patients selection and inclusion

Patients were recruited at the Neurological Unit of Pisa University Hospital. During the baseline visit, they underwent a comprehensive neurological and neuropsychological evaluation to exclude the occurrence of other neurological disorders and assess the severity of cognitive decline. The Clinical Dementia Rating scale (CDR) (Hughes et al. 1982) and Mini-Mental State Examination (Folstein et al. 1975) were used as measures of global functioning and cognition, respectively. MCI subjects were required to score CDR<1 at T0, and only mild ADD patients were included (CDR<2). A trained and certified rater performed the neuropsychological assessment, exploring the following domains: a) working memory and executive function; b) memory; c) processing speed and attention. The specific tests used were: a) Digit Span (Orsini et al. 1987), Corsi Block Span (Orsini et al. 1987), Phonemic Fluency Test (controlled oral word fluency to the letters F-A-S) (Carlesimo et al. 1996), Stroop test (Caffarra et al. 2002), and Trail Making Test (TMT) subitem B-A (Giovagnoli et al. 1996); b) Rey Auditory Verbal Learning Test (RAVLT) (Carlesimo et al. 1996), Free and Cued Selective Reminding Test (FCSRT) (Girtler et al. 2015), and Rey–Osterrieth Complex Figure Test (Carlesimo et al. 2002); c) TMT subitem A (Giovagnoli et al. 1996) and Digit Symbol Substitution Test (Lang et al. 2013). Amnestic (or hippocampal) syndrome was identified based on the pathological results for both RAVLT and FCSRT, following current research diagnostic criteria (Carlesimo et al. 1996; Albert et al. 2011; Girtler et al. 2015). Upon the execution of Brain 3T MRI, we ruled out the occurrence of severe ischemic encephalopathy (Fazekas modified scale score > 2 (Fazekas et al. 1987)) or other intra/extra-parenchymal pathologies; furthermore, we strengthened the clinical diagnosis based on cortical atrophy maps (see Results section of this S1). Other exclusion criteria included severe cardiac or psychiatric comorbidities, alcohol and/or substance abuse, and severe hepatic/renal/metabolic/endocrine diseases.

Voxel-based morphometry and hippocampal volume

For voxel-based morphometry (VBM) analysis the data were analyzed with an optimized VBM protocol (Good et al. 2001)carried out with FSL tools (http://fsl.fmrib.ox.ac.uk/fsl). The structural images were segmented and registered to the 2mm MNI 152 standard space to create a study-specific grey matter template. Then all our GM images were non-linearly register to the study-specific template and concatenate them into a 4D image. A design matrix for a general linear model was constructed in FSL to compare grey matter differences between two-group (accounting age as a nuisance covariate). TFCE-based (Threshold-Free Cluster Enhancement) analysis was applied with 10000 permutations, family-wise error rate (FWE) was controlled and FWE-corrected p-values less than 0.05 were accepted. For the hippocampal volume analysis method, we defined the masks based on Harvard-Oxford Cortical Structural atlas. GM volumes of the left and right hippocampus were extracted for each mask and for each patient from the 4D image, and then standardized for the total intracranial volume (TIV).

Supplementary Results

Cortical morphometry and hippocampal volume

In the VBM analysis, we found that the cortical volume of the bilateral superior frontal gyri and of the middle and lower left frontal gyri were lower ADD group when compared to cMCI, but this did not reach the statistical significance ($p<0,1$) (Supplem. Figure 1). On the other hand, when we compared the ADD with the ncMCI group, the volume of several cortical areas was significantly lower than in the former group ($p<0.01$) (Supp. Figure 1). No difference was found comparing the cMCI and ncMCI groups (Supplem. Figure 1).

Baseline hippocampal volume was not different among diagnostic groups (See Supplem. Table 2). A significant reduction of hippocampal volume was found both in all subjects ($p=0.015$ for the right hippocampus and $p=0.003$ for the left one) and in cMCI ($p=0.011$ and $p=0.027$ for the right and left hippocampus, respectively). At the follow-up assessment, both cMCI and ADD showed lower hippocampal volume than ncMCI, both in the right ($p=0.002$ and $p=0.007$, respectively) and in the left ($p=0.001$ and $p=0.042$, respectively) hippocampus (Supplem. Table 1).

References

Albert MS, DeKosky ST, Dickson D, et al (2011) The diagnosis of mild cognitive impairment due to Alzheimer's disease: Recommendations from the National Institute on Aging-Alzheimer's Association workgroups on diagnostic guidelines for Alzheimer's disease. Alzheimer's & Dementia 7:270–279. https://doi.org/10.1016/j.jalz.2011.03.008

Caffarra P, Vezzadini G, Dieci F, et al (2002) Una versione abbreviata del test di Stroop: dati normativi nella popolazione italiana. Nuova Riv Neurol 12:111–115

Carlesimo G, Buccione I, Fadda L, et al (2002) Standardizzazione di due test di memoria per uso clinico: breve racconto e figura di Rey. Nuova Riv Neurol 12:1–13

Carlesimo GA, Caltagirone C, Gainotti G (1996) The Mental Deterioration Battery: normative data, diagnostic reliability and qualitative analyses of cognitive impairment. The Group for the Standardization of the Mental Deterioration Battery. Eur Neurol 36:378–384. https://doi.org/10.1159/000117297

Fazekas F, Chawluk JB, Alavi A, et al (1987) MR signal abnormalities at 1.5 T in Alzheimer's dementia and normal aging. American Journal of Roentgenology 149:351–356. https://doi.org/10.2214/ajr.149.2.351

Folstein MF, Folstein SE, McHugh PR (1975) “Mini-mental state”: A practical method for grading the cognitive state of patients for the clinician. J Psychiatr Res 12:189–198. https://doi.org/10.1016/0022-3956(75)90026-6

Giovagnoli AR, Del Pesce M, Mascheroni S, et al (1996) Trail making test: normative values from 287 normal adult controls. Ital J Neurol Sci 17:305–309. https://doi.org/10.1007/bf01997792

Girtler N, De Carli F, Amore M, et al (2015) A normative study of the Italian printed word version of the free and cued selective reminding test. Neurological Sciences 36:1127–1134. https://doi.org/10.1007/S10072-015-2237-7/TABLES/5

Good CD, Johnsrude IS, Ashburner J, et al (2001) A voxel-based morphometric study of ageing in 465 normal adult human brains. Neuroimage 14:21–36. https://doi.org/10.1006/NIMG.2001.0786

Hughes CP, Berg L, Danziger W, et al (1982) A New Clinical Scale for the Staging of Dementia. The British Journal of Psychiatry 140:566–572. https://doi.org/10.1192/BJP.140.6.566

Lang M, Michelotti C, Bardelli E (2013) WAIS-IV: Weschsler Adult Intelligence Scale IV, lettura dei risultati e intepretazione clinica. RC Editore Ed.

Orsini A, Grossi D, Capitani E, et al (1987) Verbal and spatial immediate memory span: normative data from 1355 adults and 1112 children. Ital J Neurol Sci 8:539–548. https://doi.org/10.1007/bf02333660
