# Supplementary material for: The degeneration of locus coeruleus occurring during Alzheimer’s disease clinical progression: a neuroimaging follow-up investigation
Source: Brain Struct Funct. 2024 Apr 16;229(5):1317–25. doi: 10.1007/s00429-024-02797-1 (PMC11147916; doi:10.1007/s00429-024-02797-1)
Supplement: Supplementary file 8 — Supplementary Material 8 [file 429_2024_2797_MOESM8_ESM.pdf]

Supplementary Figure 1. Voxel based morphometry

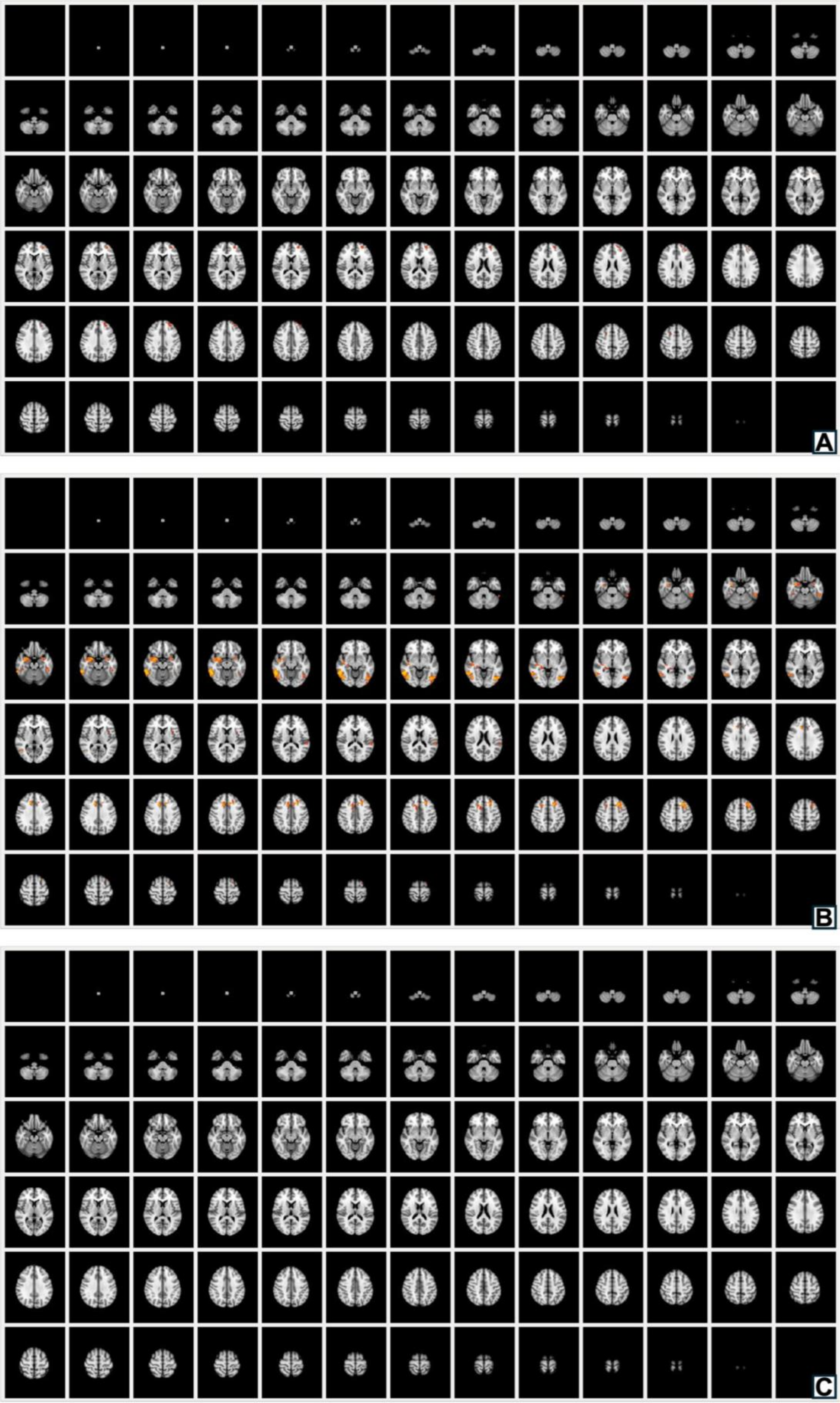

Legend to Supplementary Figure1.

(A) ADD vs cMCI groups. ADD subjects showed lower cortical volume in the bilateral frontal gyri and in the middle and inferior left frontal gyri, without statistical significance (Significance level set at $p<0.001$). Please note that to show the non-significant trend, we reported highlighted areas are significant for $p<0.1$. **(B)** ADD vs ncMCI groups. ADD subjects showed lower cortical volume in several areas (Significance level set at $p<0.001$). The statistically significant areas are here reported. In the left hemisphere: inferior temporal gyrus, temporal pole, lateral occipital cortex, central opercular cortex, parietal operculum cortex, cingulate gyrus, superior and middle frontal gyrus. In the right hemisphere: amygdala, cerebral white matter, hippocampus, temporal fusiform cortex, inferior temporal gyrus, middle temporal gyrus, lateral occipital cortex, cingulate gyrus, superior frontal gyrus. **(C)** ncMCI vs cMCI groups. No difference was observed between the two groups (Significance level set at $p<0.001$)
